# Guide to endoscope selection for peroral endoscopic myotomy procedures

**DOI:** 10.1016/j.vgie.2025.10.006

**Published:** 2025-10-17

**Authors:** Mark Kowalczyk, Robert Bechara, Fady Youssef, Rena Yadlapati, Syed Abbas Fehmi

**Affiliations:** 1 Division of Gastroenterology and Hepatology, University of California San Diego, La Jolla, California, USA; 2 Queen's University, Kingston Health Sciences Center, Kingston, Ontario, Canada

## Abstract

**Background and Aims:**

Peroral endoscopic myotomy (POEM) is the standard of care for achalasia treatment. Although prior studies have addressed technique, electrosurgical settings, and accessories, there is limited guidance on endoscope selection. This review highlights key characteristics, advantages, and limitations of commonly used endoscopes for POEM.

**Methods:**

Endoscopes from 3 major vendors were reviewed and compared across several parameters: distal outer diameter (OD), accessory channel size and position, working length, tip angulation, and ergonomics.

**Results:**

Scopes with a 3.2-mm accessory channel demonstrated greater suction capacity than 2.8-mm channels when accessory tools are in place. Vendor 1 (Olympus, Center Valley, Pa, USA) offers a slim pediatric colonoscope with a 3.2-mm channel and high maneuverability, maintaining a comparable OD to other scopes. Newer diagnostic endoscopes from vendor 2 (Fujifilm, Tokyo, Japan), including slim designs with 7.9-mm OD and 3.2-mm channels, provide enhanced suction and tip control.

**Conclusions:**

Various endoscopes are suitable for POEM, each with specific tradeoffs. Slim colonoscopes offer better maneuverability but may limit device compatibility. Intermediate-length scopes and newer 3.2-mm diagnostic scopes improve function and flexibility. Endoscopy units performing third-space procedures should consider adopting diagnostic scopes with 3.2-mm channels, which may represent a future standard of care.

## Background and Aims

Esophageal peroral endoscopic myotomy (POEM) is standard of care as a procedure for treatment of achalasia.^1^ There are multiple studies and educational resources discussing the optimal approach (eg, anterior vs posterior), electrosurgical unit settings, and accessory tools for POEM.^2^, ^3^, ^4^ Various endoscopes can be effectively used for POEM. However, to our knowledge, there currently are no resources discussing the choice of endoscope to use. Our goal is to provide a comprehensive review of common endoscope choices in North America from various popular vendors, along with their advantages and disadvantages.

## Consideration of endoscopes

**Figure 1:**
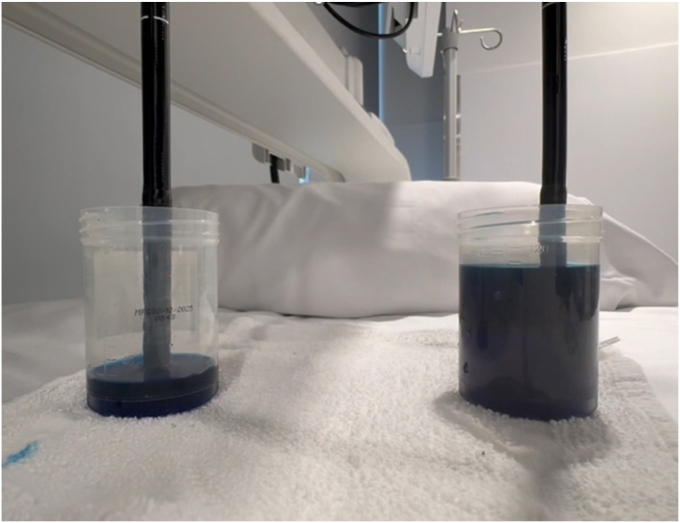
Suction of 3.2-mm channel scope (left) compared with a 2.8-mm channel scope (right) with hemostatic forceps device in both channels.

There are multiple factors one needs to consider when choosing an endoscope to use for POEM (Video 1, available online at www.videogie.org). The distal outer diameter (OD) of the scope may impact maneuverability within the submucosal tunnel, tunnel entry site size and width, and scope pushability and stiffness. One of the most important characteristics of an endoscope is the size of the accessory channel. Scopes with a 3.2-mm channel have much superior suction capability compared with a 2.8-mm channel; this is most apparent when there are accessory tools in the channel (Fig. 1.) This becomes valuable in cases of bleeding, or those with significant fibrosis requiring more injection into the submucosa during tunneling and subsequent pooling of fluid. Although there are therapeutic scopes with larger (3.7-mm) accessory channels made by various vendors, these are not commonly used for POEM procedures given larger OD and insertion tube size. The location of the channel also plays a pivotal role in tunneling, myotomy, and ergonomics of dissection. The working length of the scope may limit which accessory tools can be used. Tip angulation affects maneuverability and is particularly important in cases with atypical anatomy such as a severely dilated esophagus or sharp angulations near the gastroesophageal junction (ie, sigmoid esophagus). Additionally, lateral tip deflections can make procedures faster for endoscopists who prefer to torque less and use dials more during tunneling. Finally, the scope's length, weight, and size also are important ergonomic considerations.

## POEM accessory tools

Distal disposable attachments are important tools to use during POEM, and they create distance between the distal tip of the scope and the lumen, as well as provide traction. There are multiple vendors that make various flat plastic caps that come in different sizes to accommodate most scopes. Some flat caps have a 4-mm working distance with a small side hole to facilitate fluid exit from the cap. There are also tapered-tip caps that have a 7-mm tip length; these may have the advantage of facilitating entry into and maneuverability within the tunnel.

Accessory tools such as injector needles, knives, hemostatic forceps, and hemostatic clips are typically compatible with all endoscopes. Some accessories, for example, some versions of Speedboat (Creo Medical, Chepstow, South Wales, UK), work better with a larger accessory channel. However, there are certain tools that may not be compatible with slim colonoscopes because of inadequate length, for example, the Olympus TriangleTipKnife J and the 5- and 6-mm hemostatic forceps. Newer slim endoscopes and intermediate-length slim colonoscopes eliminate this disadvantage.

## Vendor 1

**Figure 2:**
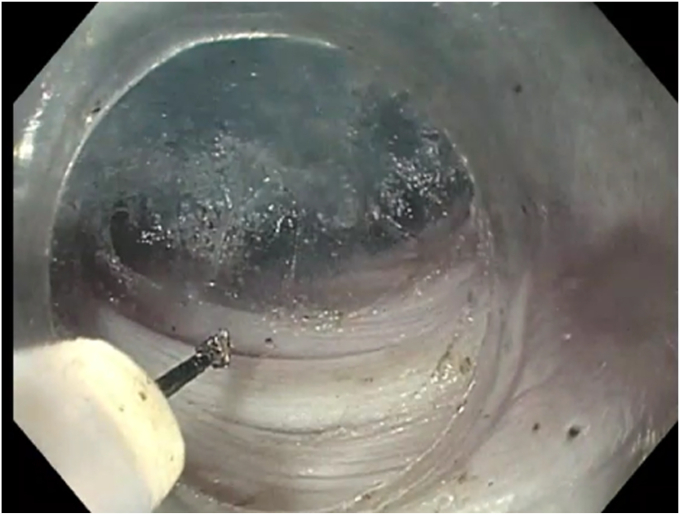
Tunneling with 9.2-mm-outer-diameter diagnostic scope from vendor 1.

Vendor 1 (Olympus, Center Valley, Pa, USA) makes diagnostic endoscopes that are commonly used for POEM given their relatively smaller distal and insertion tube ODs (Table 1). The diagnostic endoscopes come with 3 different OD sizes: 8.9, 9.2, and 9.9 mm; all have a 2.8-mm accessory channel (Fig. 2). These scopes are compatible with all caps and standard accessory tools. The device comes out at 7 o’clock; for anterior approaches, tunneling can potentially be easier and safer because the knife is aimed at the muscle layer. The shorter length of the scope is more ergonomically friendly and allows for more efficient device passage into the scope. We recommend using this scope in the setting of a narrow-caliber esophagus. Although this is likely the most commonly used type of scope for POEM, it may not be optimal for cases with anticipated bleeding, fibrosis, or a tortuous esophagus, or in cases where pushability is important.

**Table 1:** Characteristics of endoscopes from vendor 1

Model	Distal OD, mm	Insertion tube OD, mm	Working length, cm	Accessory channel size, mm	Accessory channel location, o'clock	Tip angulation: up/down/left/right, degrees
GIF-H190	9.2	9.2	103	2.8	7	210/90/100/100
GIF-HQ190	9.9	9.9	103	2.8	7	210/90/100/100
GIF-1100	8.9	8.9	103	2.8	7	210/90/100/100
PCF-H190TL	9.8	10.5	168	3.2	5	210/180/160/160
PCF-H190TI	9.8	10.5	133	3.2	5	210/180/160/160

*OD*, Outer diameter.

**Figure 3:**
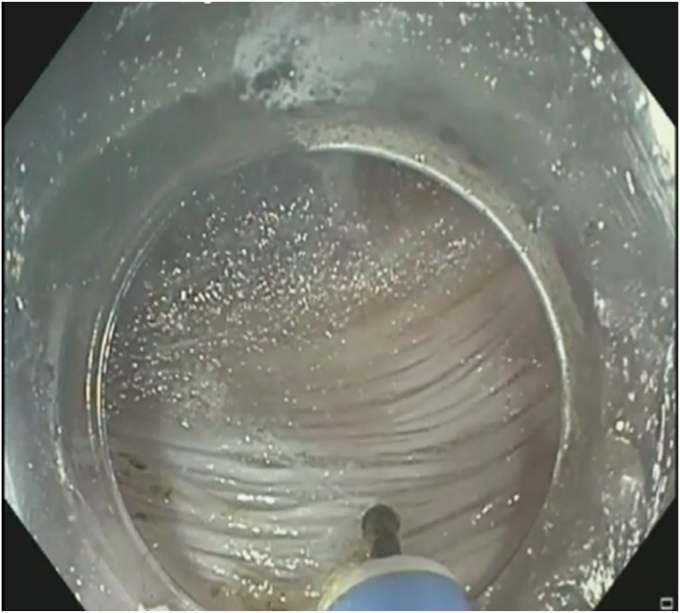
Tunneling with slim pediatric colonoscope from vendor 1.

In addition to diagnostic endoscopes, vendor 1 also makes 2 versions of a slim pediatric colonoscope that has a similar distal OD (9.8 mm OD) to the diagnostic gastroscopes but a larger insertion tube (10.5 mm OD) (Fig. 3). One key advantage of this scope is the superior tip angulations in the up, down, left, and right directions of 210°, 180°, 160°, and 160°, respectively. The length of the scope may preclude usage of certain accessory tools, and the excess length outside the patient may be bothersome to some endoscopists during the procedure. The larger 3.2-mm suction channel is particularly valuable, especially in situations with bleeding. The 5-o’clock channel location with a 6-o’clock water jet is useful in posterior tunneling and myotomy when encountering pooling of water posteriorly, and the location is also helpful in optimizing knife functioning and efficiency with scope torquing. This scope may be more optimal for cases requiring more pushability and maneuverability, as well as for situations with anticipated bleeding or fibrosis. One drawback is less optimal ergonomics given scope length, weight, and dial size. The intermediate-length scope eliminates some of these disadvantages by allowing many upper endoscope compatible length accessories to be used.

## Vendor 2

**Figure 4:**
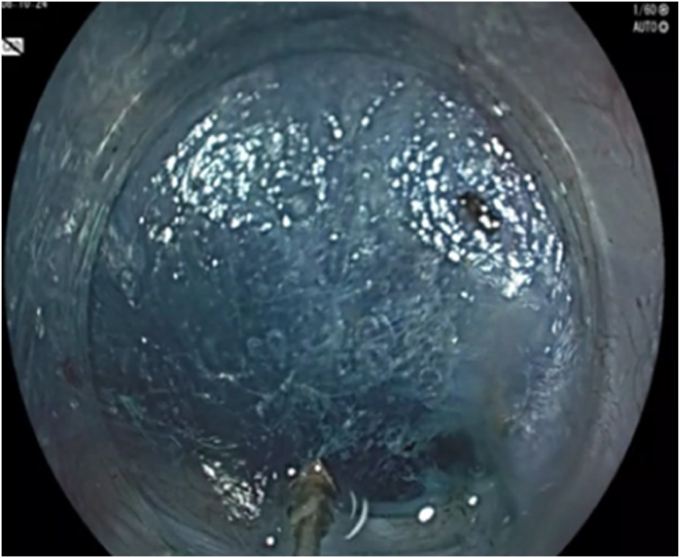
Tunneling with standard 9.2-mm-outer-diameter diagnostic scope from vendor 2.

Vendor 2 (Fujifilm, Tokyo, Japan) offers a few different endoscopes for use in POEM (Table 2). The traditional model features a distal end, shaft diameter, length, and channel size that is comparable to upper endoscopes from vendor 1 (Fig. 4). The accessory channel is positioned closer to the 6-o'clock axis—similar to that of a slim pediatric colonoscope from vendor 1. This alignment enhances knife efficiency and control during torquing or dissection. As with vendor 1's diagnostic scopes, this model is well suited for patients with a narrow-caliber esophagus. The optical characteristics differ slightly from those of other vendors because of differences in tone, sharpness, and brightness of imaging in standard settings, and user preference will play a role in determining its favorability.

**Table 2:** Characteristics of endoscopes from vendor 2

Model	Distal OD, mm	Insertion tube OD, mm	Working length, cm	Instrument channel size, mm	Accessory channel location, o’clock	Tip angulation: up/down/left/right, degrees
EG-760R	9.2	9.3	110	2.8	6-7	210/90/100/100
EG-840T	9.8	9.8	110	3.2	6-7	210/160/100/100
EG-840 TP	7.9	7.9	110	3.2	6-7	210/160/100/100

*OD*, Outer diameter.

**Figure 5:**
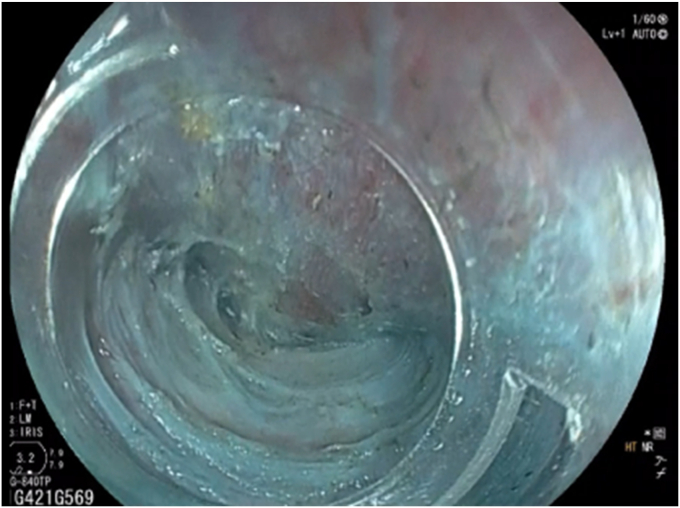
Narrow tunnel with new slim 7.9-mm-outer-diameter diagnostic scope from vendor 2 with a larger 3.2-mm channel.

Vendor 2 also offers 2 new diagnostic endoscopes including a 9.8-mm-OD version and a slim version featuring a reduced OD of just 7.9 mm (Fig. 5). This slimmer profile allows for a smaller mucosal incision during tunnel entry, enhanced maneuverability, and easier navigation, particularly through the gastroesophageal junction. It also creates a narrower dissection tunnel, which can be advantageous in select cases. Both scopes include a 3.2-mm working channel that enables effective suction even while a device is present in the channel. To our knowledge, this 7.9-mm scope represents the thinnest-caliber endoscope currently available with a channel of that size. Another standout improvement is the enhanced downward flexion of 160° that significantly aids tunnel entry and overall maneuverability during procedures. However, there are some tradeoffs. The reduced stiffness and pushability of the slim scope can lead to increased looping, which may limit its utility in cases involving a dilated esophagus, where maintaining directional control within the tunnel is critical. Overall, the refinements in vendor 2's newer diagnostic scope offer clear advantages, particularly for POEM.

## Vendor 3

**Figure 6:**
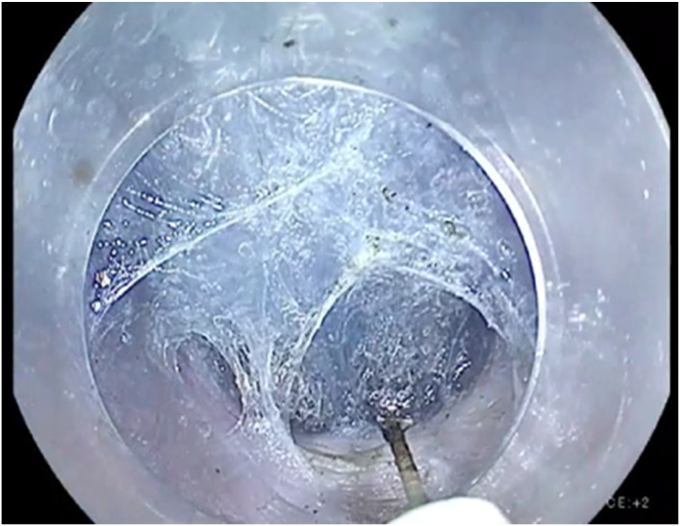
Tunneling with standard 9.9-mm-outer-diameter diagnostic scope from vendor 3.

Key features to note regarding the endoscopes from vendor 3 (Pentax, Tokyo, Japan) are the 3.2-mm instrument channel with the 9.9-mm distal OD scope, as well as the 5 o'clock channel location (Table 3, Fig. 6). Additionally, the downward deflection ability is slightly enhanced compared with the standard endoscopes from vendor 1, but inferior to their slim colonoscopes and vendor 2's new endoscopes. The tip angulation in the left and right directions of 120° is superior to all other gastroscopes, but not as high as that of the slim colonoscope.

**Table 3:** Characteristics of endoscopes from vendor 3

Model	Distal OD, mm	Insertion tube OD, mm	Working length, cm	Instrument channel size, mm	Accessory channel location, o’clock	Tip angulation: up/down/left/right, degrees
EG29-i20c	9.9	9.8	105	3.2	5	210/120/120/120
EG27-i20c	9.2	9.2	105	2.8	5	210/120/120/120

*OD*, Outer diameter.

## Discussion

There are a variety of endoscopes that can be used for POEM, each with different advantages and disadvantages (Table 4). Factors to consider when choosing which upper endoscope to use for POEM procedures may include indication of procedure, esophageal anatomy, accessory channel size, channel location, and tip flexibility.

**Table 4:** Comparison of scopes from different vendors

Model	Distal OD, mm	Insertion tube OD, mm	Working length, cm	Accessory channel size, mm	Accessory channel location, o’clock	Tip angulation: up/down/left/right, degrees	Depth of field, mm / field of view, degrees
GIF-H190	9.2	9.2	103	2.8	7	210/90/100/100	2-100/140
GIF-HQ190	9.9	9.9	103	2.8	7	210/90/100/100	2-100/140
GIF-1100	8.9	8.9	103	2.8	7	210/90/100/100	2-100/140
PCF-H190 TL	9.8	10.5	168	3.2	5	210/180/160/160	2-100/140
PCF-H190TI	9.8	10.5	133	3.2	5	210/180/160/160	2-100/140
EG-760R	9.2	9.3	110	2.8	6-7	210/90/100/100	2-100/140
EG-840T	9.8	9.8	110	3.2	6-7	210/160/100/100	2-100/140
EG-840 TP	7.9	7.9	110	3.2	6-7	210/160/100/100	2-100/140
EG29-i20c	9.9	9.8	105	3.2	5	210/120/120/120	2-100/140
EG27-i20c	9.2	9.2	105	2.8	5	210/120/120/120	2-100/140

*OD*, Outer diameter.

Endoscopes with a 3.2-mm accessory channel have superior suction ability with accessory tools in the scope, which is a very important safety consideration. Endoscopy units with processors from vendor 1 may only have the option of slim colonoscopes if they want a scope with a 3.2-mm working channel. Slim colonoscopes have enhanced tip maneuverability, but have some limitations with device compatibility and ergonomics; however, intermediate-length colonoscopes eliminate some of these disadvantages by allowing standard accessory tools to be used with the relatively shorter length.

The new diagnostic scopes from vendor 2 including the 9.2-mm OD scope and the 7.9-mm-OD slim endoscope, with both having 3.2-mm accessory channels, offer both relatively high maneuverability and superior suctioning. The 7.9-mm scope in particular appears to offer practical advantages in POEM in terms of maneuverability and tunnel navigation. Although there are currently no objective data directly comparing clinical outcomes with standard-diameter endoscopes, theoretical benefits include improved scope handling in a narrow or tortuous esophagus, potentially shorter procedure time, and reduced mucosal trauma. Future prospective studies are needed to evaluate whether these perceived advantages translate into quantifiable improvements in technical success, procedural metrics, or adverse event rates.

One limitation of our article is that we only discuss vendors and scopes primarily used in North America. Additionally, other types of scopes such as disposable endoscopes, specifically the Ambu single-use disposable gastroscope (Ambu USA, Columbia, Md, USA), have been described for use in POEM.^5^ Although these scopes share similar features to reusable scopes in terms of device channel size, flexibility, and stiffness, they may have advantages from an ergonomic standpoint because of lighter weight. Further research and experience are needed to understand how these scopes compare to reusable scopes from the vendors mentioned for POEM.

If having difficulty with 1 type of scope, one can consider the merits or demerits of other scopes to help troubleshoot a situation or to improve procedure efficiency. In our opinion, units doing third-space procedures should consider investing in diagnostic scopes with 3.2-mm working channels as they may become standard of care in the future because of improved safety compared with scopes with a 2.8-mm channel.

## Disclosures

The following authors disclosed financial relationships: R. Bechara: Consultant for Olympus and Vantage. R. Yadlapati: Consultant for Phathom Pharmaceuticals, StatLinkMD, Braintree Pharmaceuticals, Reckitt Benckiser Healthcare Ltd, Medtronic; advisory board for RJS Mediagnostix. All other authors disclosed no financial relationships.

## Supplementary data

Video 1 Guide to endoscope selection for POEM procedures.
